# Assessing professional competence in optometry – a review of the development and validity of the written component of the competency in optometry examination (COE)

**DOI:** 10.1186/s12909-020-02417-6

**Published:** 2021-01-06

**Authors:** S. Backhouse, N. G. Chiavaroli, K. L. Schmid, T. McKenzie, A. L. Cochrane, G. Phillips, I. Jalbert

**Affiliations:** 1 School of Medicine, Deakin University, Geelong, Australia; 2 Australian Council for Educational Research, Melbourne, Australia; 3 School of Optometry and Vision Science, Queensland University of Technology, Brisbane, Australia; 4 Optometry Council of Australia and New Zealand, Melbourne, Australia; 5 Department of Optometry and Vision Sciences, Melbourne School of Health Sciences, The University of Melbourne, Melbourne, Australia; 6 School of Optometry and Vision Science, The University of Auckland, Auckland, New Zealand; 7 School of Optometry and Vision Science, UNSW Sydney, Sydney, Australia

**Keywords:** Competence, Examination, Optometry, Rasch analysis

## Abstract

**Background:**

Credentialing assessment for overseas-educated optometrists seeking registration in Australia and New Zealand is administered by the Optometry Council of Australia and New Zealand. The aim was to review the validation and outcomes of the written components of this exam to demonstrate credentialing meets entry-level competency standards.

**Methods:**

The Competency in Optometry Examination consists of two written and two clinical parts. Part 1 of the written exam comprises multiple choice questions (MCQ) covering basic and clinical science, while Part 2 has 18 short answer questions (SAQ) examining diagnosis and management. Candidates must pass both written components to progress to the clinical exam. Validity was evaluated using Kane’s framework for scoring (marking criteria, item analysis), generalization (blueprint), extrapolation (standard setting), and implications (outcome, including pass rates). A competency-based blueprint, the Optometry Australia Entry-level Competency Standards for Optometry 2014, guided question selection with the number of items weighted towards key competencies. A standard setting exercise, last conducted in 2017, was used to determine the minimum standard for both written exams. Item response theory (Rasch) was used to analyse exams, produce reliability metrics, apply consistent standards to the results, calibrate difficulty across exams, and score candidates.

**Results:**

Data is reported on 12 administrations of the written examination since 2014. Of the 193 candidates who sat the exam over the study period, 133 (68.9%) passed and moved on to the practical component. Ninety-one (47.2%) passed both the MCQ and SAQ exams on their first attempt. The MCQ exam has displayed consistently high reliability (reliability index range 0.71 to 0.93, average 0.88) across all 12 administrations. Prior to September 2017 the SAQ had a set cutscore of 50%, and the difficulty of the exam was variable. Since the introduction of Rasch analysis to calibrate difficulty across exams, the reliability and power of the SAQ exam has been consistently high (separation index range 0.82 to 0.93, average 0.86).

**Conclusions:**

The findings from collective evidence support the validity of the written components (MCQ and SAQ) of the credentialing of the competency of overseas-educated optometrists in Australia and New Zealand.

**Supplementary Information:**

The online version contains supplementary material available at 10.1186/s12909-020-02417-6.

## Background

Credentialing or professional licensing is a key regulatory activity of many professional bodies. It serves many functions, including protection of the public, protection of a profession’s scope of practice, and is an important means for individuals to enter a profession and gain employment [1]. One of the key applications of the credentialing processes is to ascertain the competence and suitability of professionals trained outside a particular jurisdiction, when those competencies are not automatically recognised by virtue of the granting institution [2–4]. Credentialing assessments aim to ensure applicants are able to provide healthcare that meets the competency standards in the country they are seeking registration, while meeting workforce requirements, facilitating global mobility of practitioners, and safeguarding both the public and the professions [5, 6].

**Fig. 1:**
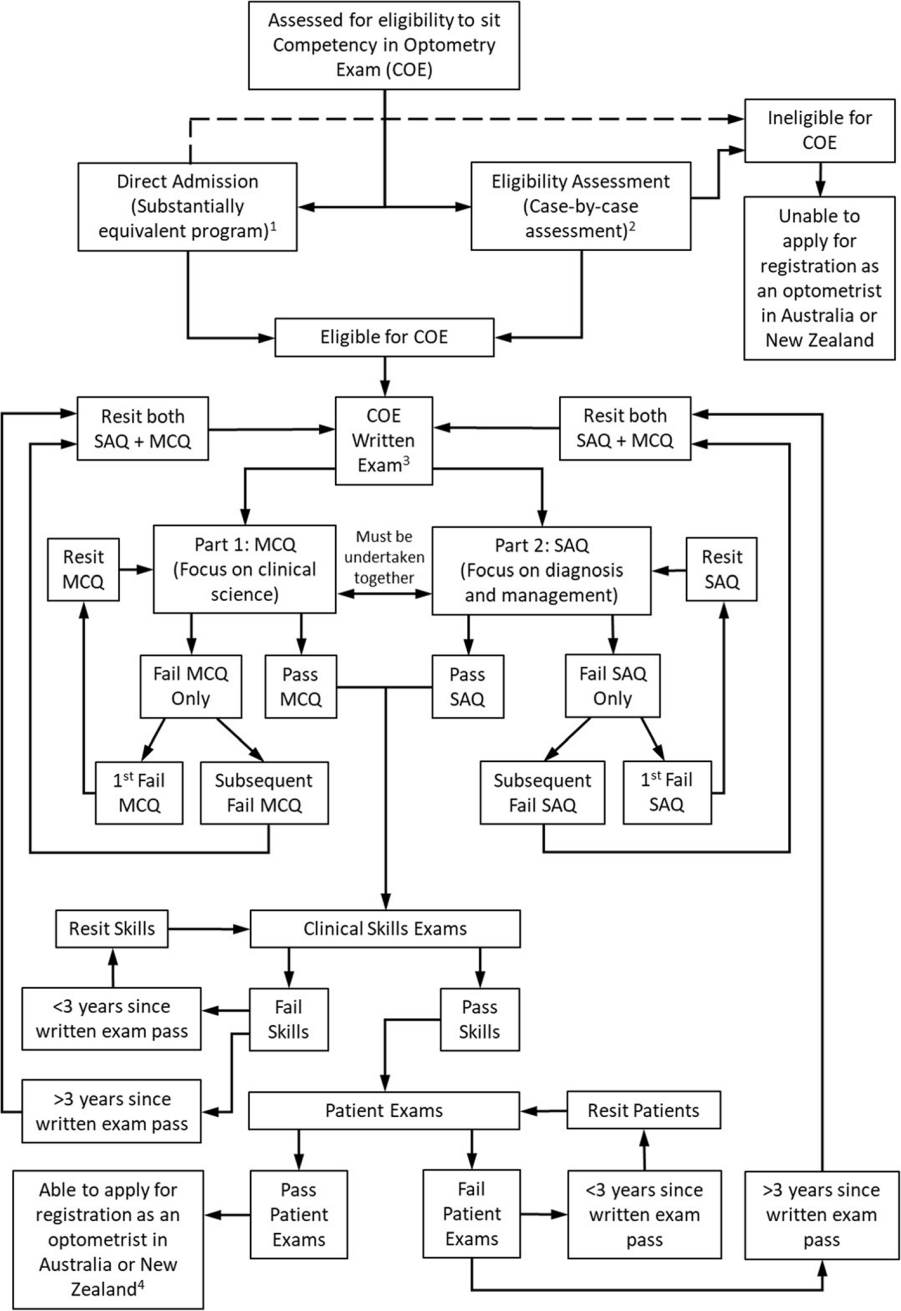
Flow diagram showing the entry pathways for overseas-trained optometrists to undertake the Competency in Optometry Exam (COE) and the possible outcomes. ^1^A full list of eligible qualifications can be found at http://www.ocanz.org/examination/competency-in-optometry-examination. ^2^To be eligible to sit the COE an applicant must provide evidence of successful completion of a course of 4 years’ full-time study (including at least one equivalent full time academic year, spent primarily in direct contact with patients to experience and learn about clinical practice, including diagnosis and management of patients), or a course of 3 years’ full-time study followed by one year supervised clinical practice after which a professional examination is passed. ^3^The multiple choice question (MCQ) and short answer question (SAQ) examinations are undertaken in the same sitting over two consecutive days. ^4^Limited registration with the Optometry Board of Australia (OBA) and provisional registration with the Optometrists and Dispensing Opticians Board in New Zealand (ODOB). The Assessment of Competence in Ocular Therapeutics (ACOT) examination or an accredited program in ocular therapeutics must then be completed within specified timeframes to gain full registration

The Competency in Optometry Examination (COE) is one such credentialing examination that is administered by the Optometry Council of Australia and New Zealand (OCANZ). OCANZ is a not-for-profit company tasked with protecting the eye health of the Australian and New Zealand public by applying quality standards to local optometry education and training in these two countries, as well as assessing overseas trained optometrists against competency standards. OCANZ has been conducting the COE twice-yearly since 1997. The COE tests the ability of overseas trained optometrists to meet the Optometry Australia Entry-level Competency Standards for Optometry [7], excluding the specific skills to be endorsed for ocular therapeutics, which are assessed separately in the Assessment of Competence in Ocular Therapeutics (ACOT) examination or an accredited program in ocular therapeutics following successful completion of the COE. The COE examination comprises two written examinations, a competency-based assessment of clinical skills and patient consultation examinations (Fig. 1). Candidates who pass the COE are eligible to apply for limited registration with the Optometry Board of Australia (OBA) and provisional registration with the Optometrists and Dispensing Opticians Board in New Zealand (ODOB).

Eligible candidates are required to pass the written examination before being able to proceed to the clinical section of the examination (Fig. 1). The written examination comprises two examination papers. The first paper, a multiple-choice question examination (MCQ) focuses on clinical science. The second paper, a short-answer question examination (SAQ) focuses on diagnosis and management. The written examination is held face to face simultaneously, over two consecutive days, at up to eight invigilated venues in Australia and overseas depending on candidate demand. At their first attempt candidates must sit both the clinical science examination MCQ and the diagnosis and management examination SAQ at the one sitting. If one of the two papers is failed, the candidate will have one further opportunity to repeat only the failed paper at a second sitting. If at the second sitting they fail that paper again, they will need to re-sit both papers at their next and at any subsequent attempts. There is no limit to how many times an eligible candidate can sit the written examination.

Entry-level competency standards for optometry in Australia were first developed in 1993 [8], and have been revised and updated in 1997 [9], 2000 [10], 2008 [11] and 2014 [7]. These competency standards are examined in, and inform the development of, all components of the COE. In this paper we describe the development, continual improvement and validation processes that have been undertaken by OCANZ to ensure the COE appropriately addresses the above competencies and credentials overseas-trained optometrists to practice in Australia and New Zealand.

Validity is a core requirement in any assessment, but perhaps especially in credentialing testing where the stakes are high and the consequences significant. Credentialing assessments must be conducted in a transparent, defensible and equitable manner in order to produce results that are reliable, valid and fair. While exam reliability can be measured quantitatively [12] and fairness can be evaluated against widely accepted criteria [13, 14], validity is best conceptualized as a qualitative judgement, presented as a reasoned argument based on evidence from multiple sources [15]. For many years validity was regarded as an inherent property of a test; the test characteristics were usually assessed through reference to psychometric indicators such as reliability coefficients, test means and pass rates [16]. This approach ignores the context and potential consequences of the test. A test might be valid for some purposes and contexts, but not for others. Accordingly, modern test validity theory now focuses on the use and interpretation of the test [17, 18]. Rather than a test being inherently valid or non-valid, it is the use and application of the test that needs to be evaluated. While psychometric indicators can still provide valuable information about a test, these are now seen as just part of the validation process, which in contemporary approaches is depicted as a form of argument or claim about the decisions or inferences which are based on the results of a particular test.

Several frameworks have been developed to operationalise such an approach to validation. Two of the most influential in the field of health practice are those developed respectively by Messick [16] and Kane [17]. Messick’s framework organises the potential sources of validity evidence into five categories, namely: content, internal structure, relationships with other variables, response process and consequences [16]. Kane’s framework goes beyond sources of evidence to represent the types of inferences which reflect the overall argument and chain of reasoning, namely: scoring, generalisation, extrapolation and implications [17]. While either approach can offer a useful framework for contemporary validation processes, Kane’s approach provides a particularly useful way of prioritising and situating the different forms of evidence within the overall argument and inferential structure [19]. In this framework, attention is given to a competency-based argument that involves linking test scores to statements about competence, and then to conclusions about expected performance in practice [20]. Furthermore, decisions about licensure or certification within this framework are based on expected performance in practice [21].

Accordingly, in this paper we organise and present the relevant evidence pertaining to the OCANZ assessment process based on Kane’s approach. The primary aim of this paper is to review and describe the validation process of one example of a credentialing examination in the health professions field. This information should be useful for other health professionals developing a credentialing system utilising written examinations as part of their process.

## Methods

According to each level of Kane’s framework [19], the relevant arguments for a written examination such as the COE include reference to the following components of the test development:

i. Scoring: e.g. choice of item format and scoring method; process of item development and peer review for determination of correct response (MCQ) and marking guide (SAQ); marker training and calibration; item analysis and descriptive statistics of candidate performance; resolution of any marker discrepancies.
ii. Generalisation: e.g. representativeness of the test in relation to the OCANZ competencies (i.e. the test blueprint); relative weightings of items across the relevant domains; the internal consistency of the results, including reliability coefficient and item correlations.
iii. Extrapolation: e.g. authenticity of tasks and the relationship between a test result and real-world proficiency, and/or performance on other related measures; the acceptability and appropriateness of the designated minimum standard (as reflected in the pass mark).
iv. Implications: e.g. broader or ‘downstream’ effects or impact of the test, such as pass rates, subsequent performance of successful candidates on the next stage of assessment, and ultimate impact on patient safety and clinical performance in optometric practice; appropriate consideration of impact on candidates including cost, transparency, preparation requirements and support.

Such considerations are presented and discussed in greater detail below, as part of the validation process for the use of the COE exam for determining minimum acceptable competence of overseas-trained optometrists for practice in Australia and New Zealand.

### Examination content and constructs

The written component of the COE consists of two exams: an MCQ exam focused on clinical science and an SAQ exam focused on diagnosis and management (Fig. 1). The MCQ exam contains 144 items to be answered in 180 min, consisting of 120 scored questions and 24 non-scored ‘pilot’ questions undergoing validation for future examinations. (Prior to 2018, the MCQ examination consisted of 132 questions, 12 of which were non-scored questions.) The candidates are not made aware of which questions are scored and which are non-scored. All MCQ items are written in the single best answer format, consisting of a ‘stem’, a single correct answer (‘key’), and three incorrect options (‘distractors’). Candidates are instructed to determine the single response that best answers the question.

The MCQ exam assesses candidates’ foundational knowledge of basic biomedical, vision, optical, and clinical sciences, along with their ability to apply this knowledge in a clinical scenario. The questions vary between knowledge recall questions, to more contextual questions that rely on clinical reasoning and integration. The questions for each exam are drawn from an item bank containing approximately 600 questions. The questions in the bank have been written over the course of 12 years by subject matter experts commissioned by OCANZ. Pre-existing questions in the item bank were blueprinted against the 2008 competencies [11]. The questions in the bank were re-blueprinted in 2017/2018 to cover the appropriate clinical science competencies from the revised Optometry Australia Entry-level Competency Standards for Optometry 2014 [7] that were adopted in 2017. A list of the 2014 competencies that are assessed, and the approximate number of questions from each competency found in a typical MCQ exam, can be found in Additional file 1: Appendix 1.

The SAQ exam consists of 18 short answer questions to be completed in 180 min. Each question carries an equal weighting, is scored out of 10 marks (with half-marks), and is scored to a marking rubric. The SAQ exam has been designed to test the candidates’ higher level diagnostic decision making processes [22, 23], in conjunction with the foundational knowledge assessed in the clinical science MCQ exam. Candidates are variously required to: describe abnormal or normal features; discuss observations in anatomical, biochemical, microbiological and/or pathological terms; offer a diagnosis or diagnoses to account for observations and provide justifications for the diagnoses; suggest appropriate treatment or management, including criteria for referral or monitoring; and list systemic, ocular and visual signs and symptoms associated with the condition. The SAQ questions consist of multiple parts based upon short clinical vignettes, often accompanied by a photograph of the clinical condition. Unlike the MCQs that are blueprinted against a single competency per item, the SAQs assess candidates simultaneously across multiple competencies. A list of the 2014 competencies that are assessed, and the approximate number of questions from each competency found in a typical SAQ exam, can be found in Additional file 1: Appendix 2.

All examination scripts are anonymised for marking, with only a candidate identification number provided to the optometry school contracted to undertake the marking. Individual questions are marked by the same examiner across all candidates, and papers from individual candidates that are judged as ‘borderline’ are reviewed by an independent marker. The raw score for each question is used for psychometric analysis as outlined in detail below. Prior to 2018 the marking of the SAQ scripts was rotated amongst the optometry education providers in Australia and New Zealand, but since then, following an open call for expressions of interest, it has been assigned to a single optometry school to promote marker consistency and assessment expertise.

### Quality assurance and standard setting

New items are regularly produced and specifically commissioned when areas are identified as having insufficient cover in the item bank as a result of changing professional competencies. All item writers are trained by an educational expert prior to producing items. A formal Question Writing Guide was commissioned to establish approved guidelines for the writing of new items. New items are reviewed, and edited where necessary, by the Written Sub-committee of the OCANZ Examination Committee, then piloted as non-scored items to validate them before they are included in the question bank as scored items. Any questions that perform poorly as a pilot item are reviewed by the Written Sub-committee and either discarded or edited and re-piloted.

The entire COE process was externally audited by an experienced educational consultant in 2015/2016. This review concluded that the COE was consistent with international best practice for this type of credentialing examination, with minor improvements suggested around the examination processes.

The examination cutscores are determined through a formal process of standard setting. The minimum passing standard was re-established at a workshop led by an experienced psychometrician and attended by content experts from Australia and New Zealand in February 2017. Two standard setting approaches were used for the MCQ items based on the two most recently administered exams: 30 items from the September 2016 exam were assessed using a modified Angoff procedure with Beuk and Hofstee adjustment; 26 items from the April 2016 exam were assessed using a bookmark method [24]. The cutscores derived from the standard setting exercise were highly correlated with the cutscores that were implemented in the April and September 2016 exams, thereby validating the cut-score locations. The SAQ items were standard set using nine questions from the September 2016 exam using an Angoff borderline rating method. Follow-up standard setting workshops will be held every 3–4 years to ensure appropriate maintenance of the OCANZ scale and cut-scores for the COE.

### Psychometric analysis of results

Excel Psychological and Educational Consultancy Pty Ltd. (EPEC; https://www.epecat.com) are contracted by OCANZ to perform psychometric analysis of the COE written exam results. The Rasch model, which estimates and takes into account individual question difficulty and person ability, is used to determine cutscores based on variations in these from one exam to another (reviewed in [25]).

The MCQ results are analysed using both classical statistics and Rasch analysis. In order to maintain comparable cutscores over different exams, a linking and equating process is performed through the use of 20 common items (out of the 120 scored items) between successive examination papers. The calculated difficulty estimates and errors of the common items in the exam being analysed are standardised and adjusted to equate them to the common items in the previous exam paper. Chi-square significance is calculated for each of these linking items, and if any item falls outside of the 95% confidence bands it is removed from the exam calibration. The difficulties of the remaining items are anchored to the results of the previous exam, and the cutscore is set such that the candidate ability required to pass is maintained across examinations.

Rasch analysis is also undertaken on the SAQ scores following a log transformation to achieve equality of intervals on a logit scale given the polytomous nature of the raw data for each question. As for the MCQ examination, the SAQ contains two common, linking questions from the previous exam, and along with content expert standard setting scores this enables equating of the cutscores to maintain the same required candidate performance levels for a pass across exams. Any questions from the MCQ or SAQ exam that are identified as performing poorly through the Rasch analysis are referred back to the content experts in the Written Examination Sub-committee for checking prior to final result release.

### Ethics and data statement

This was a retrospective analysis of the outcomes of the written examinations based on data collected by OCANZ over the preceding 6 years. The research was conducted in accordance with the tenets of the Declaration of Helsinki and was approved by The University of Melbourne, the School of Health Sciences Human Research Ethics Committee. A waiver was granted as written informed consent was not able to be retrospectively obtained from the candidates that had sat the examinations. The data are not publicly available due to it containing information that could compromise the privacy of the examinees or the integrity of the credentialing examination. The data was deidentified such that individual examinees privacy could not be compromised. The authors declare that the aggregated data supporting the findings of this study are available within the article.

## Results

### Candidate results and demographic data

Data from 6 years of administration of the COE, from 2014 to 2019, were analysed for this report, representing 12 administration rounds in total. There were 193 candidates who sat at least one component of the exam during this period, with 272 total administrations over the 6 years as a result of some candidates sitting multiple times (see Fig. 1 for progression pathways). There were 133 candidates (68.9%) who passed both the MCQ and SAQ components of the COE and moved on to the practical component across the six-year period (12 administrations) analysed. A detailed analysis of the candidate results and demographic data can be found in Additional file 1: Appendix 3.

### MCQ exam performance

**Fig. 2:**
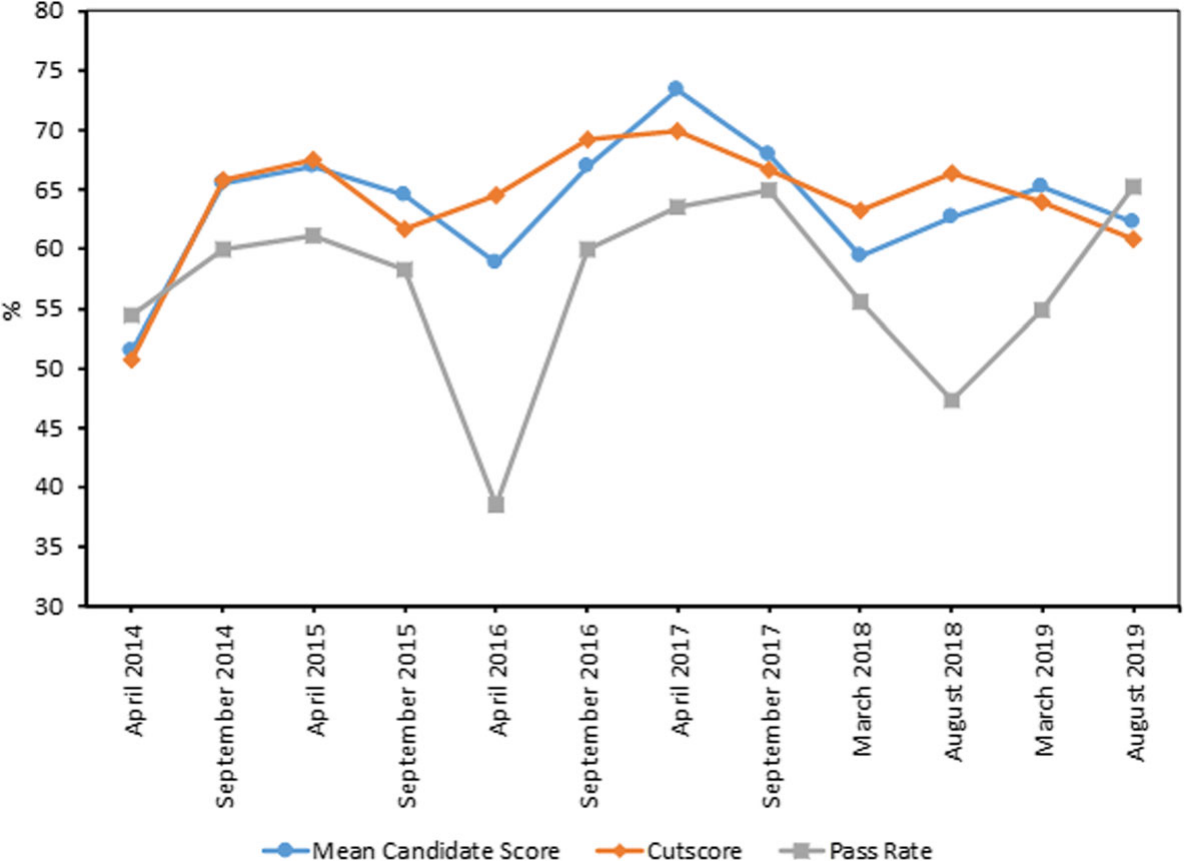
Mean candidate scores and corresponding cutscores and pass rates for each administration of the multiple choice question (MCQ) component of the Competency in Optometry Exam (COE)

As a consequence of the linking and equating undertaken as part of the Rasch analysis of the results the overall difficulty of the exam has remained relatively stable across administrations, with mean candidate scores ranging from 51.5 to 73.3% (Table 1 and Fig. 2). The MCQ exam has displayed highly consistent reliability, with an average reliability index of 0.88 across 12 administrations (range 0.71 to 0.93; Table 1).

**Table 1:** Descriptive statistics for all multiple choice question (MCQ) exam administrations

Administration Round	1	2	3	4	5	6	7	8	9	10	11	12
Examination date	April 2014	September 2014	April 2015	September 2015	April 2016	September 2016	April 2017	September 2017	March 2018	August 2018	March 2019	August 2019
No. of scored questions (total no. of questions)	120 (132)	120 (132)	120 (132)	120 (132)	120 (132)	120 (132)	120 (132)	120 (132)	120 (144)	119^c^ (144)	119^c^ (144)	120 (144)
No. of linking questions	23	23	20	19	19	21	26	18	10	19	12	16
No. of candidates	33	25	18	24	13	20	22	20	18	19	22	23
No. of repeat candidates	14	15	5	10	4	4	7	5	4	8	4	8
Mean candidate score (%)	51.5	65.6	66.9	64.5	58.9	66.9	73.3	68.0	59.4	62.7	65.2	62.2
Max total score (%)	74	81	83	86	70	83	82	89	78	81	79	78
Min total score (%)	34	49	44	33	43	37	61	38	36	30	49	32
Mean item facility (%)	– ^d^	– ^d^	66.8 ± 21.6	64.5 ± 19.7	58.9 ± 23.7	66.9 ± 18.1	73.0 ± 21.4	68.0 ± 20.2	59.3 ± 19.2	62.7 ± 19.1	65.2 ± 21.5	62.2 ± 23.0
Mean discrimination index^a^	0.2^e^	0.2^e^	0.23 ± 0.24	0.26 ± 0.20	0.18 ± 0.33	0.26 ± 0.24	0.17 ± 0.24	0.33 ± 0.20	0.25 ± 0.28	0.29 ± 0.24	0.19 ± 0.23	0.29 ± 0.24
Reliability index^b^	0.80	0.79	0.85	0.88	0.78	0.89	0.71	0.93	0.89	0.91	0.79	0.91
Cutscore (%)	50.8	65.8	67.5	61.7	64.6	69.2	70.0	66.7	63.3	66.4	63.9	60.8
Pass rate (%)	54.5	60.0	61.1	58.3	38.5	60.0	63.6	65.0	55.6	47.4	54.9	65.2

Descriptive statistics for every MCQ administration for all candidates between 2014 and 2019. (^a^) Mean value for the discrimination index calculated for each item as part of the classical analysis undertaken by EPEC. (^b^) Reliability index alpha (KR-20) calculated for the exam as part of the classical analysis undertaken by EPEC. A reliability index greater than 0.80 is considered high paper reliability. (^c^) One scored item excluded from analysis following content expert review after being flagged by the preliminary Rasch analysis. (^d^) Detailed individual item reports not requested from EPEC consultancy firm prior to April 2015 administration. (^e^) Detailed individual item reports not available from EPEC consultancy firm prior to April 2015 administration. The values reported here represent the mean discrimination index provided by EPEC from the classical analysis, rather than an analysis of the individual item point biserial values, therefore no standard deviations showing the spread of the data are available.

**Fig. 3:**
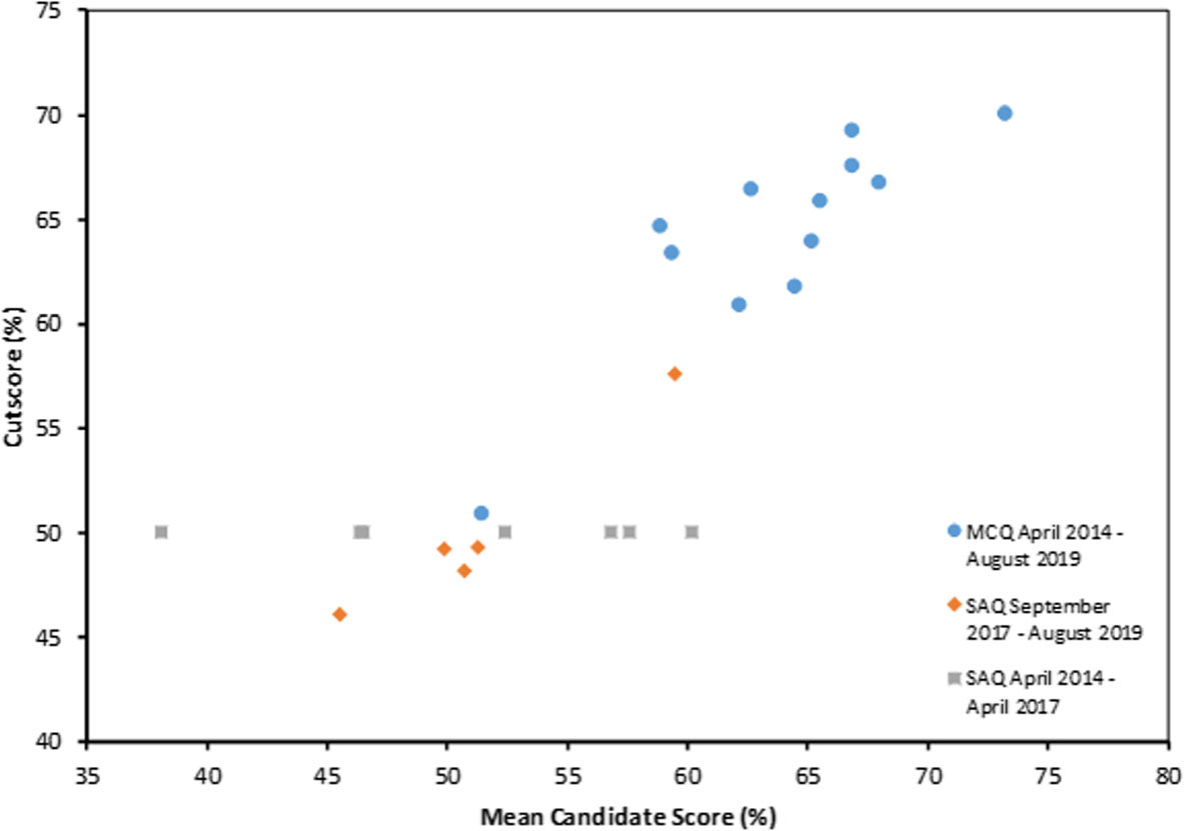
Correlation between candidate performance and cutscore for the multiple choice question (MCQ) and short answer question (SAQ) exams. A strong relationship was observed for the MCQ exams (r^2^ = 0.74, *p* < 0.001) and the SAQ exams from September 2017 to August 2019 (r^2^ = 0.95, *p* = 0.005). The SAQ exams from April 2014 to April 2017 used a standard cutscore of 50%. The high correlation for the SAQ exams from September 2017 to August 2019 highlights the benefit of using Rasch analysis to set the cutscore compared to the 50% cutscore used previously

The relationship between cutscores and candidate performance is fairly consistent, with increases in the cutscore mirroring increases in the mean candidate score (Figs. 2 and 3). A strong correlation between the MCQ mean candidate score and the cutscore was observed (r^2^ = 0.74, *p* < 0.001). No correlation was observed, however, between the pass rate and the cutscore (r^2^ = 0.02, *p* = 0.627). This is possibly the result of low pass rates in April 2016 (38.5%), when there were only 13 candidates, and August 2018 (47.4%), when 8 out of 19 candidates were repeating, suggesting pass rate is influenced by a cohort effect.

### SAQ exam performance

**Fig. 4:**
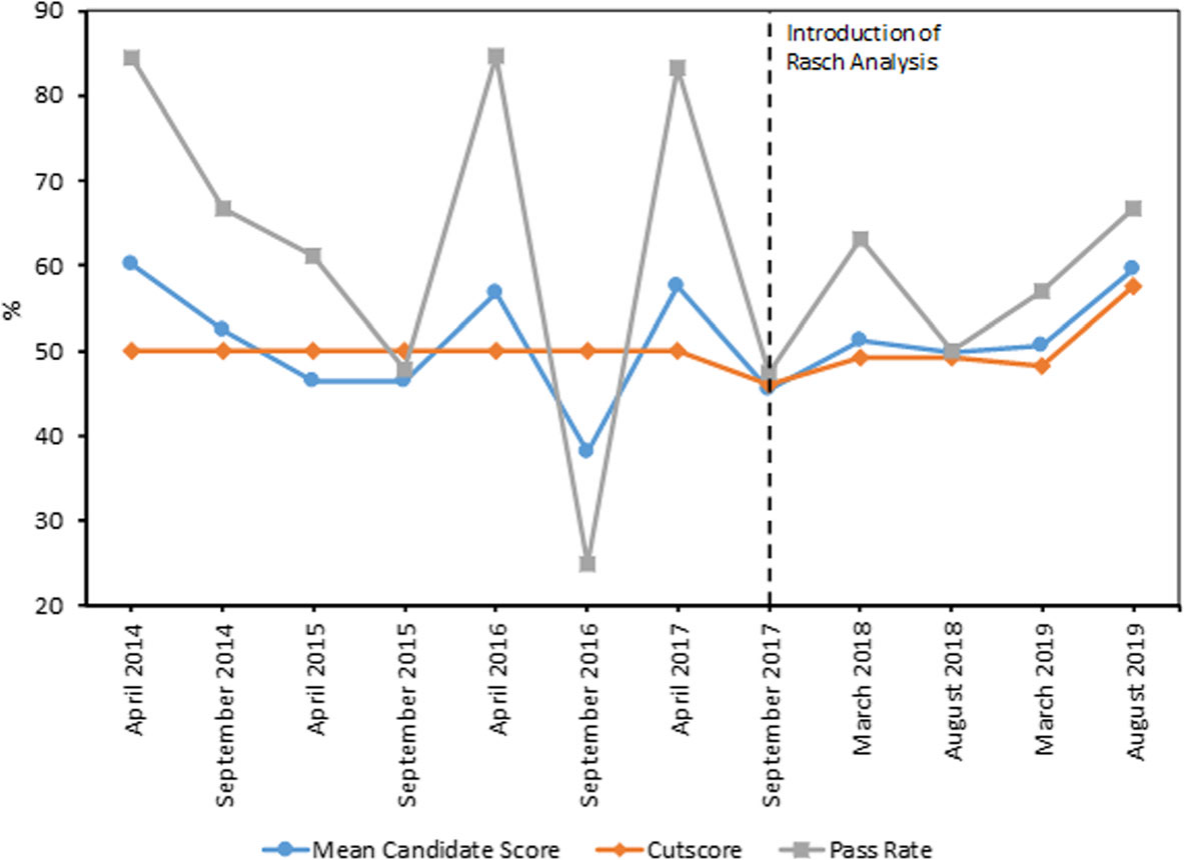
Mean candidate scores and corresponding cutscores and pass rates for each administration of the short answer question (SAQ) component of the Competency in Optometry Exam (COE). Rasch analysis was introduced from September 2017 to set the cutscore and analyse the exam. Prior to this a cutscore of 50% was used

Prior to the September 2017 offering of the SAQ exam Rasch analysis was not conducted, linking questions not included and the cutscore was set at 50% for all administrations. As such the overall difficulty of the SAQ exam prior to September 2017 was more variable compared to the exams after the introduction of Rasch analysis (Table 2 and Fig. 4). Across all 12 administrations the mean candidate scores ranged from 38.1 to 60.2%. Prior to September 2017 the mean scores ranged from 38.1 to 60.2%, while the mean scores from September 2017 onwards ranged from 45.5 to 59.5% showing less variability (Fig. 4). Since the introduction of Rasch analysis, the SAQ exams have displayed consistent reliability and good power, with an average separation index of 0.86 across five administrations (range 0.82 to 0.93; Table 2). The questions in the SAQ since September 2017 have been well targeted to the abilities of the candidates, with mean person abilities averaging around zero logits. The SAQ exams also closely fit the Rasch model with Chi-square item-trait interaction probabilities that are highly insignificant. These data all indicate that the reliability of the SAQ exam has improved following the introduction of Rasch analysis in September 2017 compared to the greater variability seen with a 50% cutscore in earlier iterations.

**Table 2:** Descriptive statistics for all short answer question (SAQ) exam administrations

Administration Round	1	2	3	4	5	6	7	8	9	10	11	12
Examination date	April 2014	September 2014	April 2015	September 2015	April 2016	September 2016	April 2017	September 2017	March 2018	August 2018	March 2019	August 2019
No. of scored questions^a^	18	18	18	18	18	18	18	18	18	18	18	18
No. of candidates	32	21	18	23	13	16	24	19	19	18	21	24
No. of repeat candidates	12	11	5	9	4	1	9	4	5	7	3	9
Mean candidate score (%)	60.2	52.4	46.4	46.5	56.8	38.1	57.6	45.5	51.3	49.9	50.7	59.5
Max total score (%)	84.0	70.3	58.0	60.5	67.8	57.1	67.8	63.6	68.9	70.8	68.3	71.9
Min total score (%)	24.4	26.9	22.0	8.0	42.8	5.0	42.5	18.6	27.8	30.0	31.9	31.4
Mean person ability (logits)^b^	– ^e^	– ^e^	– ^e^	– ^e^	– ^e^	– ^e^	– ^e^	0.154	−0.068	−0.122	0.233	0.102
Item-trait interaction (χ^2^ probability)^c^	– ^e^	– ^e^	– ^e^	– ^e^	– ^e^	– ^e^	– ^e^	0.93	0.42	0.39	0.76	0.42
Reliability (separation index)^d^	– ^e^	– ^e^	– ^e^	– ^e^	– ^e^	– ^e^	– ^e^	0.93	0.89	0.84	0.83	0.82
Cutscore (%)	50^f^	50^f^	50^f^	50^f^	50^f^	50^f^	50^f^	46.1	49.3	49.2	48.2	57.6
Pass rate (%)	84.4	66.7	61.1	47.8	84.6	25.0	83.3	47.4	63.2	50.0	57.1	66.7

Descriptive statistics for every SAQ administration for all candidates between 2014 and 2019. (^a^) Each question consists of multiple sections, adding to a total of 10 marks per question (scored with half marks) and 180 marks per exam. Two questions were used for linking exams from September 2017 onwards. (^b^) Mean person ability, in logits, from the Rasch analysis undertaken by EPEC consultancy on behalf of the Optometry Council of Australia and New Zealand (OCANZ) for the Competency in Optometry Exam (COE). The low mean person abilities indicate that the questions are well targeted to the candidates. (^c^) The item-trait interaction gives a measure of whether the data fits the Rasch model for discreet groups (class intervals) along the scale. χ^2^ values above 0.05 indicate the data do not significantly deviate from that expected from the model. (^d^) Separation index values from the Rasch analysis undertaken by EPEC consultancy on behalf of OCANZ for the COE. These reliability values being greater than 0.8 show good fit of the Rasch model to the scores, indicating the SAQ exams have good power and reliability. (^e^) Rasch analysis of the SAQ exam not undertaken prior to the September 2017 running of the exam, therefore no mean person ability or separation index values available to report. (^f^) Rasch analysis of the SAQ exam not used to calculate a cutscore until the September 2017 running of the exam. Prior to September 2017 the cutscore was set as 50%.

The relationship between cutscores and candidate performance in the SAQ following the introduction of Rasch analysis is very consistent, with increases in the cutscore mirroring increases in the mean candidate score (Figs. 3 and 4). A strong correlation between the SAQ mean candidate score and the cutscore was observed (r^2^ = 0.95, *p* = 0.005). As with the MCQ exams, no correlation was observed between the pass rate and the cutscore (r^2^ = 0.60, *p* = 0.126).

### MCQ item analysis

The selection of MCQ items for each exam is based on desired weightings for each of the competencies examined in the MCQ exam (see Additional file 1: Appendix 1). Since April 2015, 1198 MCQ questions have been administered across ten 120 item exams (two items were removed from scoring following expert review, see Table 1), with most questions having been administered in more than one exam. Across all 1198 questions the average item facility was 64.8 ± 21.1%, with an average discrimination index of 0.24 ± 0.25 across 1056 of these questions (items with either 0% or 100% correct responses having no discrimination index). When the items were grouped by competency, the mean facilities (range 56.3 to 94.8%) and discrimination indices (range 0.18 to 0.32) indicated that all competencies were being assessed at the appropriate level by the MCQ items, and performing as expected in separating the stronger candidates from those with less knowledge. There were no competencies that candidates performed extremely poorly on, and only competencies that had very few cumulative question administrations displayed mean facilities above 80%. Overall this suggests that the MCQ is well balanced and is achieving the desired outcome of assessing the clinical science focus appropriately.

Each SAQ exam question addresses more than one competency (see Additional file 1: Appendix 2 for the approximate weighting of each competency across all SAQ questions in each exam). It is therefore not possible to separate out and analyse the individual performances of each competency with the SAQ items as has been undertaken for the MCQ items.

## Discussion

Credentialing of health professionals trained outside of a particular jurisdiction is an important regulatory function for many professional bodies. A number of different approaches to credentialing have been used by a wide range of health professions [2–4]. A core requirement of credentialing assessment is the validity of the assessment. This paper described the approach that OCANZ took with the COE to credential overseas trained optometrists within Kane’s validity framework [17]. The use of Kane’s framework to understand each of the key components of the test development (Scoring, Generalisation, Extrapolation and Implications [19]) has resulted in a valid, repeatable and feasible written exam. The use of Rasch analysis alongside Kane’s framework led to an improved understanding of the exam metrics, the quality of individual questions and comparison of exam difficulty from one administration to the next [17, 25], ensuring the assessment was both valid and reliable [12].

Individual candidate performance was not dependent on sex, age, or years since graduation, but did vary with the region where the primary optometry degree was obtained. The outcomes of the linking questions were used to determine exam difficulty for each administration and vary the cut-scores used to determine the pass mark if required. Cut scores for the MCQ varied from 50.8 to 70% and for the SAQ from 46.1 to 57.6%. This represents relatively small variations in exam difficulty that were accounted for in determination of the score required by candidates to show competency with the content. The reliability of the MCQ exam ranged from 0.71 to 0.93 (mean 0.84) but was 0.78 or greater for all but one exam. This represents acceptable to good reliability [26]. Separation index for the SAQ ranged from 0.82 to 0.93 (mean 0.87) confirming that the SAQ was able to distinguish candidates in terms of their ability on this exam. Separation index values greater than 0.8 indicate that there is good fit of the Rasch model to the scores, which confirms the SAQ exams have good power and reliability [26]. The analysis shows that the exams were able to discriminate candidates on their knowledge and that variations in exam difficulty were accounted for. As candidates may pass one exam and fail the other this suggests the MCQ and SAQ exams are testing different competencies and that both exams types are important measures. Candidates should have confidence in this robust and fair assessment process that means that they have the knowledge required to progress to the next stage of the COE.

Content experts were able to use the information about individual questions to replace poorly performing questions and to identify competency areas for which new questions needed to be developed. New questions were piloted before inclusion in the question bank and this ensured only high quality questions were added. Training on how to write MCQs has been shown to lead to better quality questions [27], as does peer review [28], and both of these are undertaken when producing new MCQs and SAQs for the COE.

As the skills and competencies of the profession change [7–11] the exam content will continually need to be updated to ensure it remains aligned to these, as seen in other professional exams [29, 30]. The method described here is a manageable way of ensuring an up-to-date, appropriate, content-specific exam. The analysis of content alignment to professional competencies does require a range of expertise and is relatively time intensive [31] and costly, but quality control processes are important. The quality control processes implemented by OCANZ in setting the COE gives candidates sitting the exams confidence in their outcomes. Indeed, the processes undertaken in the validation of the COE closely align with those detailed in both Kane’s framework [19], and the steps undertaken to ensure validity by other credentialing bodies [2–4]. The optometry profession and general public can be assured through the validation of the credentialing exam that overseas-trained optometrists registered in Australia and New Zealand have met the appropriate competency standards to practice.

The scopes and competencies of the practice of Optometry in Australia and New Zealand continue to expand. The challenge for the validation process of the written component of the COE is to ensure it regularly reviews itself so that its outcomes continue to reflect the changing needs of the profession. The end result of the process should be to continue to assess the ability for overseas applicants to meet contemporary Australian and New Zealand competencies, especially when these competencies may differ from those of the home country. We believe the process of change we describe reflects the evolution of the OCANZ validation process over time and the robustness of these changes.

The small number of candidates undertaking each offering of the exam is a limitation of our analysis. Despite the low participant numbers, the consistently high reliability scores, with low inter-examination variability, should give confidence that the validation of the exams is appropriate. The small candidate numbers do mean, however, that the content experts need to be cautious when assessing the reliability of individual multiple-choice questions from a single examination instance. A further limitation of the present study is that it focused solely on the written component of the credentialing examination process. Additional analysis of the clinical examinations will need to be undertaken to ensure validity of the entire COE process.

## Conclusions

The findings from collective evidence from the past 6 years of administration of the COE provide support for the validity of the written components (MCQ and SAQ) of the credentialing of the competency of overseas-educated optometrists undertaken in Australia and New Zealand. Candidates sitting the exam can have confidence in their outcomes. While the presented case is specific to the credentialing of optometrists, the processes described provide a blueprint that can be adopted by a wide range of health professions undertaking credentialing assessments.

## Supplementary Information

**Additional file 1:** **Appendix 1**

## Data Availability

The data are not publicly available due to it containing information that could compromise the privacy of the examinees or the integrity of the credentialing examination. The data was deidentified such that individual examinees privacy could not be compromised. The authors declare that the aggregated data supporting the findings of this study are available within the article.

## Abbreviations

COE: Competency in Optometry Examination
MCQ: Multiple choice question
OCANZ: Optometry Council of Australia and New Zealand
SAQ: Short answer question
