# Supplementary material for: Assessing professional competence in optometry – a review of the development and validity of the written component of the competency in optometry examination (COE)
Source: BMC Med Educ. 2021 Jan 6;21:11. doi: 10.1186/s12909-020-02417-6 (PMC7786977; doi:10.1186/s12909-020-02417-6)
Supplement: Supplementary file 1 — Additional file 1: Appendix 1 [file 12909_2020_2417_MOESM1_ESM.docx]

| **Elements** | **Specific Performance Criteria (Where Applicable)** | **Questions †** |
| --- | --- | --- |
| 1.1 Maintains, develops and audits optometric knowledge, clinical expertise and skills. | 1.1.1 Optometric knowledge, equipment and clinical skills are maintained and developed. | 1 |
| 1.2 Adopts an evidence-based approach as the foundation for making clinical decisions. |  | 2 |
| 1.5 Communicates appropriate advice and information. |  | 3.5 |
| 1.8 Understands the legal and other obligations involved in optometric practice. | 1.8.1 Relevant legislation, common law obligations relevant to practice and Australian Standards are understood and implemented. | 1.5 |
| 1.12 Understands factors affecting the community's need for eye-care services. |  | 2 |
| 2.5 Obtains, interprets and takes account of patient information from sources other than the patient. |  | 3.5 |
| 3.1 Formulates an examination plan. 3.2 Implements an examination plan. |  | 7 |
| 3.3 Assesses the ocular adnexae and the eye. |  | 18 |
| 3.7 Assesses visual information processing. |  | 2 |
| 3.8 Assesses signs and symptoms found during the ocular examination that have significance for the patient's systemic health. |  | 4 |
| 4.2 Evaluates the expected prognosis of the condition. |  | 2 |
| 4.5 Prescribes spectacles. |  | 13 |
| 4.6 Dispenses spectacle prescriptions accurately. |  | 6 |
| 4.7 Prescribes contact lenses. |  | 16 |
| 4.8 Prescribes low vision devices. |  | 6 |
| 4.9 Prescribes pharmacological and other regimes to treat ocular disease and injury. **‡** | 4.9.5 Patients are instructed about precautionary procedures and non-pharmacological and palliative management. 4.9.6 Patients are instructed in the avoidance of cross-infection. 4.9.7 Non-pharmacological treatment or intervention procedures, therapeutic device fitting and emergency ocular first aid are performed to manage eye conditions and injuries. | 12 |
| 4.10 Manages patients requiring vision therapy. |  | 3 |
| 4.11 Refers patients and receives patient referrals. | 4.11.1 The need for referral to other professionals or rehabilitative services for assessment and/or treatment is recognised, discussed with the patient and a suitable professional or service is recommended. | 4 |
| 4.13 Co-operates with ophthalmologist/s in the provision of pre- and post-operative management of patients. |  | 8 |
| 4.14 Provides advice on vision, eye health and safety in the workplace and recreational settings. |  | 3 |
| 5.2 Maintains confidentiality of patient records. |  | 1 |
| 5.3 Meets legislative requirements regarding retention and destruction of patient records and other practice documentation. |  | 1.5 |
|  | **Total Number of Questions:** | **120** |

**Appendix 1.** Table showing the Optometry Australia Entry-level Competency Standards for Optometry 2014 (modified from reference [7]) that are examined in the 120 item, single best answer, Clinical Science MCQ examination that forms the first part of the OCANZ Competence in Optometry Examination. **†**The approximate number of questions from each assessed competency that are included in a typical examination. **‡**Note that ocular therapeutics and pharmacological treatment are not assessed in the COE as candidates must either complete the Assessment in Ocular Therapeutics (ACOT) examination conducted by OCANZ (if they hold an overseas qualification in ocular therapeutics) or undertake an approved ocular therapeutics course.

| \| **Competency** \| **Specific Elements and Performance Criteria** \| **Questions †** \| \| --- \| --- \| --- \| \| Formulation of examination plan, assessment of the ocular adnexae and the eye, diagnosis \| 1.2.1 Clinical expertise is integrated with the best available evidence, the patient's perspective and the practice context when making clinical decisions. 3.1.1 An examination plan based on the patient history is designed to obtain the information necessary for diagnosis and management. 3.1.2 Tests and procedures appropriate to the patient's condition and abilities are selected. 3.2.1 Tests and procedures which efficiently provide the information required for diagnosis are performed. 3.3 Assesses the ocular adnexae and the eye. 4.1 Establishes a diagnosis or diagnoses. \| 3 \| \| Assessment of pupil function, establishment of diagnoses, interpretation and analysis of findings to establish a diagnosis, including formulation and implementation of examination plan \| 1.2.1 Clinical expertise is integrated with the best available evidence, the patient's perspective and the practice context when making clinical decisions. 3.1 Formulates an examination plan. 3.2.1 Tests and procedures which efficiently provide the information required for diagnosis are performed. 3.4.4 Pupil function is assessed. 4.1 Establishes a diagnosis or diagnoses. \| 1 \| \| Treatment/management program \| 1.2.1 Clinical expertise is integrated with the best available evidence, the patient's perspective and the practice context when making clinical decisions. 4.4 Designs a management plan in consultation with the patient and implements the agreed plan. \| 2 \| \| Prescription of contact lenses, including formulation and implementation of an examination plan \| 1.2.1 Clinical expertise is integrated with the best available evidence, the patient's perspective and the practice context when making clinical decisions. 3.1.1 An examination plan based on the patient history is designed to obtain the information necessary for diagnosis and management. 4.7.1 The suitability of contact lenses as a form of correction for the patient is assessed and discussed. 4.7.2 The patient's refraction, visual requirements and other findings are applied to determine the contact lens prescription and lens type. 4.7.4 Contact lenses with new fitting parameters are assessed on the eye prior to supply to the patient. \| 1.5 \| \| Assessment of visual fields and colour vision including examination plan and interpretation and analysis of findings to establish a diagnosis \| 1.2.1 Clinical expertise is integrated with the best available evidence, the patient's perspective and the practice context when making clinical decisions. 3.1.1 An examination plan based on the patient history is designed to obtain the information necessary for diagnosis and management. 3.4.2 Visual fields are measured. 3.4.3 Colour vision is assessed. 4.1 Establishes a diagnosis or diagnoses. \| 2 \| \| Assessment of oculomotor and binocular function including examination plan, including interpretation and analysis of findings to establish a diagnosis \| 1.2.1 Clinical expertise is integrated with the best available evidence, the patient's perspective and the practice context when making clinical decisions. 3.1.1 An examination plan based on the patient history is designed to obtain the information necessary for diagnosis and management. 3.6.1 Eye alignment and the state of fixation are assessed. 3.6.2 The quality and range of the patient's eye movements are determined. 3.6.3 The status of binocularity is determined. 3.6.4 The adaptability of the vergence system is determined. 3.6.5 Placement and adaptability of accommodation are assessed. 4.1 Establishes a diagnosis or diagnoses. \| 1.5 \| \| Significance of incidental findings/investigation of ocular signs and symptoms \| 1.2.1 Clinical expertise is integrated with the best available evidence, the patient's perspective and the practice context when making clinical decisions. 3.8 Assesses signs and symptoms found during the ocular examination that have significance for the patient's systemic health. \| 1.5 \| \| Contact lens aftercare including examination plan \| 1.2.1 Clinical expertise is integrated with the best available evidence, the patient's perspective and the practice context when making clinical decisions. 3.1.1 An examination plan based on the patient history is designed to obtain the information necessary for diagnosis and management. 4.7.6 A contact lens prescription is written in a manner that can be interpreted for correct fabrication of the appliance. \| 1 \| \| Referral of the patient/choice of practitioner for referral \| 1.2.1 Clinical expertise is integrated with the best available evidence, the patient's perspective and the practice context when making clinical decisions. 4.11.1 The need for referral to other professionals or rehabilitative services for assessment and/or treatment is recognised, discussed with the patient and a suitable professional or service is recommended. 4.11.2 Timely referral, with supporting documentation, is made to other professionals. 4.13.4 Appropriate referral for further post-operative treatment or assessment of complications is arranged. \| 1.5 \| \| Treatment of adnexal and anterior eye disorders \| 1.2.1 Clinical expertise is integrated with the best available evidence, the patient's perspective and the practice context when making clinical decisions. 4.9.1 Pharmacological agents are selected and recommended. 4.9.2 An ocular therapeutic prescription is issued in a manner that allows accurate supply of the agent. \| 1 \| \| Provision of pre- and post-operative management \| 1.2.1 Clinical expertise is integrated with the best available evidence, the patient's perspective and the practice context when making clinical decisions. 4.13.1 Pre-operative assessment and advice are provided. 4.13.2 Post-surgical follow-up assessment and monitoring of signs according to the surgeon's requirements and the procedure undertaken. 4.13.3 Emergency management for observed post-surgical complication is provided. \| 0.5 \| \| Advice on vision in the workplace \| 4.14.3 Individuals are counselled on the suitability of their vision for certain occupations. 4.14.4 Certification of an individual's visual suitability for designated occupations or tasks is provided. \| 0.5 \| \| Emergency care and eye health promotion \| 1.10 Provides or directs patients to emergency care. 1.11.2 Advice is provided on eye protection for occupational and home-based activities and for recreational pursuits. \| 1 \| \|  \| **Total Number of Questions:** \| **18** \| |  |  |
| --- | --- | --- | --- | --- | --- | --- | --- | --- | --- | --- | --- | --- | --- | --- | --- | --- | --- | --- | --- | --- | --- | --- | --- | --- | --- | --- | --- | --- | --- | --- | --- | --- | --- | --- | --- | --- | --- | --- | --- | --- | --- | --- | --- | --- | --- | --- | --- |

**Appendix 2.** Table showing the Optometry Australia Entry-level Competency Standards for Optometry 2014 (modified from reference [7]) that are examined in the 18 question Diagnosis and Management SAQ examination that forms the second part of the OCANZ Competence in Optometry Examination. **†**The approximate number of questions from each assessed competency that are included in a typical examination.

**Appendix 3**

*Candidate Results and Demographic Data*

There were 193 candidates who sat at least one component of the exam between 2014 and 2019, with 272 total administrations over the six years as a result of some candidates sitting multiple times. The demographic data for the most recent attempt at the COE for the 193 candidates is found in Appendix 3 Table 1. There were 133 candidates (68.9%) who passed both the MCQ and SAQ components of the COE and moved on to the practical component across the six-year period analysed. Ninety-one candidates (47.2%) passed both the MCQ and SAQ exams on their first attempt. The average number of attempts needed by the other 42 candidates, who did not pass on their first attempt, was 2.40 (range: 2 to 5 attempts). There were 60 candidates (31.1%) who failed their most recent attempt at the written component of the COE: 31 (16.1%) failed both the MCQ and the SAQ; 15 (7.8%) failed only the MCQ exam; 14 (7.3%) failed only the SAQ exam. Out of the 60 fails, 29 candidates (15.0%) were sitting the exam for the first time. The average number of attempts undertaken by the other 31 candidates who failed, and were repeating one or more component of the written exam, was 2.65 (range: 2 to 5 attempts).

**Appendix 3 Table 1.** Demographics, pass/fail rates, and number of attempts for candidates by the region in which they undertook their training

| **Region of Training^a^** | **No. of Candidates (% of Total)** | **No. of Passes (%^b^)** | **No. of Passes on First Attempt (%^b^)** | **Average No. of Attempts to Pass (Range)** | **No. Failed both MCQ and SAQ (%^b^)** | **No. Failed MCQ Only (%^b^)** | **No. Failed SAQ Only (%^b^)** | **No. Failed First Attempt (Yet to Resit) (%^b^)** | **Average No. of Attempts (Failing Candidates) (Range)** |
| --- | --- | --- | --- | --- | --- | --- | --- | --- | --- |
| All | 193 | 133 (68.9%) | 91 (47.2%) | 1.44 (1,5) | 31 (16.1%) | 15 (7.8%) | 14 (7.3%) | 29 (15.0%) | 1.85 (1,5) |
| Europe^c^ | 97 (50.3%) | 80 (82.5%) | 60 (61.9%) | 1.32 (1,3) | 1 (1.0%) | 8 (8.2%) | 8 (8.2%) | 10 (10.3%) | 1.76 (1,4) |
| Africa^d^ | 47 (24.4%) | 27 (57.4%) | 16 (34.0%) | 1.59 (1,5) | 13 (27.7%) | 6 (12.8%) | 1 (2.1%) | 8 (17.0%) | 1.85 (1,3) |
| Asia/Subcontinent/ Middle East^e^ | 39 (20.2%) | 20 (51.3%) | 11 (28.2%) | 1.80 (1,5) | 15 (38.5%) | 1 (2.6%) | 3 (7.7%) | 10 (25.6%) | 1.89 (1,5) |
| America^f^ | 10 (5.2%) | 6 (60.0%) | 4 (40.0%) | 1.33 (1,2) | 2 (20.0%) | 0 (0.0%) | 2 (20.0%) | 1 (10.0%) | 2.00 (1,3) |

**Appendix 3 Table 1.** Demographics, pass/fail rates, and number of attempts for all candidates, grouped by the region in which they undertook their training, who sat the written component of the COE between 2014 and 2019. When a candidate had multiple attempts at the COE during the study period, only the most recent attempt is included in the presented data. (a) Due to low numbers of candidates from some countries the data have been grouped into regions to protect the identity of individuals. (b) Percentage of the regional total. (c) Ireland, Poland, Scotland, United Kingdom. (d) Ghana, Nigeria, South Africa. (e) Hong Kong, India, Iran, Korea, Malaysia, Nepal, Pakistan, Philippines, Singapore. (f) Canada, Columbia, USA.

The number of candidates sitting the COE each administration has remained relatively stable across the 12 administrations. The average number of candidates sitting the MCQ exam was 21.4 (range: 13 to 33), with an average of 7.3 (range: 4 to 15) candidates repeating the exam each administration (Table 1). The average number of candidates sitting the SAQ exam was 20.7 (range: 13 to 32), with an average of 6.6 (range: 1 to 12) candidates repeating the exam each administration (Table 2). Around half of all candidates (n = 97; 50.3%) were from Europe, the majority being from the United Kingdom (Appendix 3 Table 1). The other regions represented in the candidate composition were: Africa (n = 47; 24.4%), with the majority from South Africa; Asia/Subcontinent/Middle East (n = 39; 20.2%), with the majority from India and Hong Kong; and America (n = 10; 5.2%), with the majority from USA. Interestingly there was a much higher pass rate across all attempts in the candidates from Europe (82.5%) compared to those candidates from America (60.0%), Africa (57.4%), or Asia (51.3%) (χ^2^ = 17.24, p < 0.001). Those candidates from Europe who failed were more likely to fail either the MCQ or the SAQ exam only, whereas candidates from other regions were more likely to fail both exams (χ^2^ = 26.46, p < 0.001). Overall, 31 out of 60 candidates who failed in their most recent attempt failed both the MCQ and SAQ exams. There was no trend towards candidates being more likely to fail the MCQ or the SAQ with 15 and 14 failing each exam respectively. This suggests that the two exams are assessing different aspects of the candidates’ knowledge, as intended by their design (a clinical science focus for the MCQ exam and a diagnosis and management focus for the SAQ exam; see Appendix 1 and 2).

The year of graduation was recorded for 103 of the candidates. The average number of years between graduating in their home country to sitting the COE was 9.2 years (range 1 to 32 years). Seventy two candidates passed both the MCQ and SAQ exams, with an average time since graduation of 8.4 years (range 1 to 32 years). The 31 candidates who failed at least one of the MCQ and SAQ exams had a slightly longer average time since graduation of 10.8 years (range 3 to 27 years). Overall, however, there was no significant difference in the average time since graduation between the candidates who passed and those who failed the COE (df = 101, p = 0.081; unpaired t-test).
